# Polymorphism in Gag Gene Cleavage Sites of HIV-1 Non-B Subtype and Virological Outcome of a First-Line Lopinavir/Ritonavir Single Drug Regimen

**DOI:** 10.1371/journal.pone.0024798

**Published:** 2011-09-20

**Authors:** Jade Ghosn, Constance Delaugerre, Philippe Flandre, Julie Galimand, Isabelle Cohen-Codar, François Raffi, Jean-François Delfraissy, Christine Rouzioux, Marie-Laure Chaix

**Affiliations:** 1 Paris Descartes University, EA 3620, Necker University Hospital, Paris, France; 2 AP-HP, Department of Internal Medicine and Infectious Diseases, Bicetre University Hospital, Le Kremlin-Bicetre, France; 3 AP-HP, Virology Department, Necker University Hospital, Paris, France; 4 Inserm UMR-S 943, Virology Department, Pitié-Salpêtrière Hospital and Pierre et Marie Curie University Paris 6, Paris, France; 5 Abbott France, Rungis, France; 6 Department of Infectious Diseases, Hotel-Dieu Hospital, Nantes, France; INSERM, France

## Abstract

Virological failure on a boosted-protease inhibitor (PI/r) first-line triple combination is usually not associated with the detection of resistance mutations in the protease gene. Thus, other resistance pathways are being investigated. First-line PI/r monotherapy is the best model to investigate *in vivo* if the presence of mutations in the cleavage sites (CS) of *gag* gene prior to any antiretroviral treatment might influence PI/r efficacy. 83 patients were assigned to initiate antiretroviral treatment with first-line lopinavir/r monotherapy in the randomised Monark trial. We compared baseline sequence of gag CS between patients harbouring B or non-B HIV-1 subtype, and between those who achieved viral suppression and those who experienced virological failure while on LPV/r monotherapy up to Week 96. Baseline sequence of gag CS was available for 82/83 isolates; 81/82 carried at least one substitution in gag CS compared to HXB2 sequence. At baseline, non-B subtype isolates were significantly more likely to harbour mutations in gag CS than B subtype isolates (p<0.0001). Twenty-three patients experienced virological failure while on lopinavir/r monotherapy. The presence of more than two substitutions in p2/NC site at baseline significantly predicted virological failure (p = 0.0479), non-B subtype isolates being more likely to harbour more than two substitutions in this specific site. In conclusion, gag cleavage site was highly polymorphic in antiretroviral-naive patients harbouring a non-B HIV-1 strain. We show that pre-therapy mutations in gag cleavage site sequence were significantly associated with the virological outcome of a first-line LPV/r single drug regimen in the Monark trial.

## Introduction

Complete viral suppression may be achieved in 64 to 84% of antiretroviral-naïve HIV-infected patients starting a ritonavir-boosted protease inhibitor based first-line combination [1]–[4]. Unlike virological failure on a first-line non-nucleoside analogue reverse transcriptase inhibitor (NNRTI)-containing regimen, failure on a first-line PI/r based triple combination is rarely associated with the detection of resistance mutations in HIV protease and reverse transcriptase genes [1], [4], [5]. Indeed, the development of PI resistance is usually a stepwise process occurring in treatment-experienced patients, with first the accumulation of major mutations leading to resistance to one or several protease inhibitors and decreasing viral fitness [6]–[8], and then, minor mutations, which partially subsequently restore viral replication [9]–[12]. The fact that failure on a first-line PI/r combination is rarely associated with the detection of resistance mutations has led to the search for other resistance mechanisms enabling HIV to become resistant to PI without modification of the viral protease. One hypothesis might be that mutations are selected outside the *protease* gene, i.e. in the *gag* gene.

The HIV protease cleaves the gag and gag-pol polyproteins by interacting with specific cleavage sites (CS) in *gag* and *pol* genes. In the product of the gag open reading frame, Gag polyproteins are cleaved at five cleavage sites into p17 (MA), p24 (CA), p2 (SP1), p7 (NC), and p6gag. In the product of the gag-pol open reading frame, Gag-Pol polyproteins are cleaved at eight cleavage sites into p17 (MA), p24 (CA), p2 (SP1), p7 (NC), transframe protein (TFP), p6pol, protease, reverse transcriptase, and integrase [13], [14]. Frameshifting is required for producing Gag and Gag-Pol polyprotein precursors in HIV-1, similar to many other retroviruses. Frameshifting is a rare controlled event, occurring only for 1 of 10 to 20 ribosomes. It is driven by the secondary RNA structure also called hairpin structure and allows for a correct Gag/Gag-Pol ratio to ensure optimal virus activity [15], [16]. The RNA folding and stability of the gag-pol frameshift region can be evaluated by the measure of hairpin free energy.

Mutations in Gag CS emerge as compensatory mutations enabling specific protease mutants to have a greater efficiency of cutting the Gag polyprotein [9], [12], [17], [18]. Interestingly, after full genomic sequencing, Nijhuis et al. reported on three viruses resistant to a novel PI without any resistance-associated mutation in *protease* gene but harbouring NC/p1 CS substitutions in the viral Gag polyprotein (K436E and or I437T/V) in [19]. This effect was driven essentially by the C-terminal region. Mutations in NC-SP2-p6 gag CS were found indeed to confer a 3- to 6-fold increase in phenotypic resistance factors to PIs and/or to enhance PI resistance conferred by mutations in the *protease* gene [20], [21]. Potential underlying mechanisms of resistance may involve an increase in the mutant protease activity by a compensatory mechanism and/or a higher level of production of protease.

Though substitutions in gag CS are detected often in PI-experienced HIV-infected patients [22], recent studies have shown that such substitutions are also evident in antiretroviral-naïve HIV-infected patients [19], [23]. Moreover, Nijhuis et al showed that these CS substitutions were highly significantly associated with reduced susceptibility to PI in clinical isolates lacking primary protease mutations [19]. Thus, in antiretroviral-naïve HIV-infected patients starting a first-line combined antiretroviral therapy (cART), the presence of CS mutations might be associated with a decreased activity of protease-containing regimens, but its impact on cART outcome might be less pronounced in a context of triple combination with two nucleoside reverse transcriptase inhibitors (NRTI).

Monark was the first randomized trial comparing the efficacy of lopinavir/r (LPV/r) single drug regimen with a classical triple combination in antiretroviral-naïve HIV-infected patients starting a first-line regimen [24], [25]. In this study, the proportion of patients achieving complete viral suppression was lower in the LPV/r single drug arm than in the triple combination arm. However, only 5 patients out of the 23 experiencing virological failure while on LPV/r drug selective pressure harbored a viral strain with major PI resistance-associated mutations [26]. First-line LPV/r monotherapy represents the ideal model to investigate whether the presence of pre-therapeutic mutations in gag CS is associated with virological failure in the absence of protease-associated resistance mutations. We therefore sequenced gag CS at baseline in all 83 viral isolates from patients randomised to LPV/r single drug regimen in the Monark trial and then compared baseline sequence of gag CS between patients achieving full virological suppression and those experiencing virological failure while on LPV/r single drug selective pressure. We also sequenced gag CS at the time of virological failure to look for additional mutations that would have been selected under drug-selective pressure.

## Methods

### Monark study design

Monark study design has been described elsewhere [24]. The study protocol was approved by the Ethics Committees in each participating country (France: Comité d'Ethique de l'Hôpital de Bicêtre; Germany: Ethik-Kommission der Aerztekammer Berlin, Ethikkommission Charité Universitätsmedizin Berlin, Ethikkommission Heinrich Heine-Universitaet Dusseldorf, Ethikkommission Bayerische Landesaerztekammer Muenchen; Spain: Comité Ético de Investigación Clínica Barcelona; Italy: Comitato Etico Brescia, Comitato Etico Torino, Comitato Etico della Fondazione Milano, Comitato Etico Locale per la Sperimentazione Clinica dell'Ospedale Luigi Sacco di Milano, Comitato EticoR Roma; and Poland: Komisja Bioetyczna Warsaw). All patients provided written informed consent. Briefly, patients were randomly assigned to receive first-line LPV/r monotherapy or LPV/r plus ZDV/3TC if they were naïve to antiretroviral therapy, had a CD4 cell count above 100/mm^3^, a plasma HIV-1 RNA below 100 000 copies/mL and no evidence of drug-resistance at screening visit. The primary endpoint was the proportion of patients with plasma HIV-1 RNA below 400 copies/mL at week 24 (W24) and below 50 copies/mL at W48. Follow up until W96 was planned for evaluation of the long-term safety and efficacy of the LPV/r monotherapy arm [25]. Sub-optimal response was defined as (i) failure to achieve a decline in viral load of at least 1.0 log_10_ copies/mL by W4, (ii) failure to achieve a viral load below 400 copies/mL by W24 and (iii) any viral rebound ≥1 log, after an HIV-1 RNA<400 copies/mL, confirmed by a second measurement at least 14 days later.

### Resistance testing

Reverse transcriptase and protease genotypic resistance tests were performed at screening and at the time of VF according to the trial definition [26]. The resistance analysis was extended also to patients with low-level viremia (between 50 and 400 copies/mL) after W24. Thirty-three patients experienced VF during the study course: 23/33 were on LPV/r single drug regimen at the time of VF and the remaining ten had discontinued study treatment. Gag resistance testing was focused on patients experiencing VF while under LPV/r drug selective pressure (n = 23). Protease inhibitor (PI) resistance mutations were defined according to 2008 IAS list (www.iasusa.org).

### Determination of viral subtype

The HIV-1 subtype was determined after phylogenetic analysis of the reverse transcriptase sequences as previously described [26]. We analyzed gag CS mutations according to subtype B and other subtypes as non B.

### Amplification and Analysis of Gag region

*Gag* genes were sequenced at baseline in all patients randomized to LPV/r monotherapy and at the time of confirmed virological failure. Viral RNA was extracted from plasma stored at −70°C using QIAamp® RNA Mini Kit (Qiagen SA, Courtaboeuf, France). Amplification and sequencing were done with primers as previously described [19]. All sequences were centralized at the Necker Virology Laboratory.

Different CS gag appellations have been used over time (the ones used in recent literature are between bracket). Differences in frequency of amino acid sequences for CS CA/p2 (or p24/p2), p2/NC (or p2/p7), NC/p1 (or p7/p1), p1/p6*gag* in the *gag* reading frame, transframe protein (TFP), TFP/p6*^pol^* and p6*^pol^*/PR in the *gag-pol* reading frame, with respect to the wild-type virus HXB2 were studied. Mixtures containing wild-type and mutant variants were scored as mutant.

### Determination of hairpin free energy

Baseline RNA folding and the stability of the hairpin structure of the gag-pol frameshift region were determined using measurement of free energy in accordance with Turner's rules (CombFold, RNAsoft [http://www.rnasoft.ca/cgi-bin/RNAsoft/CombFold/combfold.pl]).

### Statistical analysis

The distribution of gag CS mutations was described according to HIV-1 subtype B and non-B at baseline in all samples and at the time of virological failure in 23 patients. Fisher's exact test for discrete variables and Wilcoxon-rank-sum test for continuous data were used to compare groups of patients. A multivariate logistic regression was used to identify independent significant factors associated with virological outcome. The variables investigated included the presence or absence of substitutions at position A374 or V484 or S451 in gag CS at baseline, B versus non-B subtype and sub-optimal (having missed at least one dose of study treatment between baseline and Week 96) versus good adherence (no missed dose throughout follow-up).

## Results

### Baseline gag CS mutations and impact on virological response

*Gag* gene sequence was available for 82 among the 83 patients randomized to LPV/r monotherapy and followed until W96. At baseline, 81/82 isolates carried at least one substitution in gag CS compared to HXB2 sequence, with a median number of 3 (range 0–10): 4/82 isolates carried at least one substitution in CA/p2, 76/82 in p2/NC, 15/82 in NC/p1, 45/82 in p1/p6 site in the *gag* reading frame, and 80/82 in TFP/p6^pol^ and 81/82 in p6*^pol^*/PR in the *gag-pol* reading frame. Among the gag CS mutations in the *gag* reading frame previously described in therapy-experienced isolates (A431V, K436R, I437V, L449F/V, P452S, P453L/A) [23], the K436R mutation was evident at baseline in 6 patients, the I437V mutation in 2, the L449F mutation in 1 and the P453L mutation in 5 patients.

**Figure 1:**
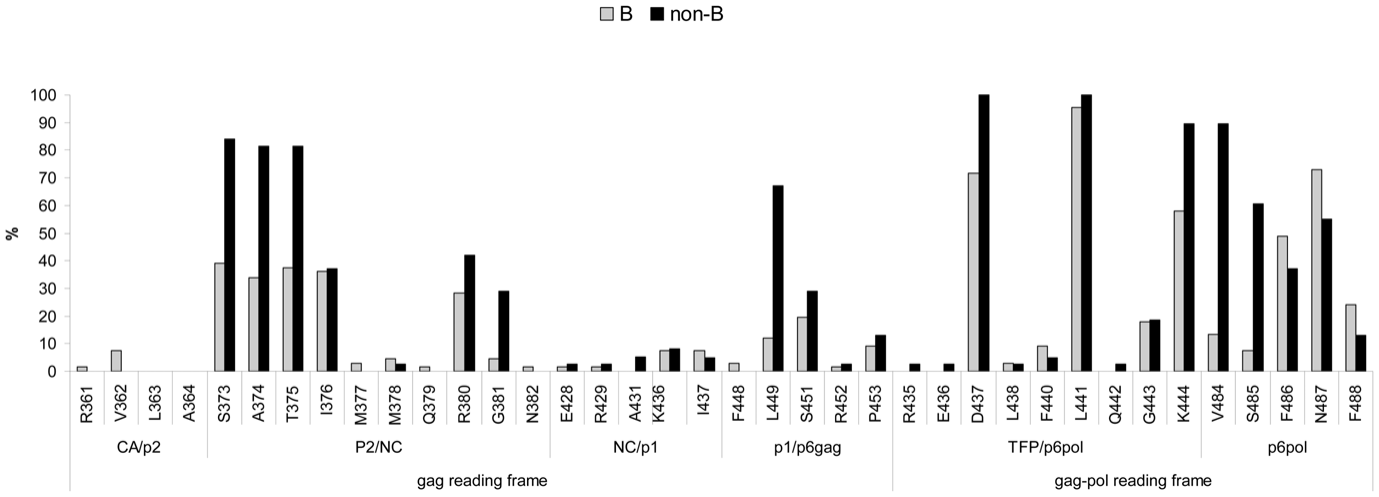
Frequency of gag cleavage site mutations at baseline according to HIV-1 B and non-B subtype. The gag cleavage sites mutations are described on the gag and the gag-pol reading frames. Frequencies of mutations are presented according to HIV-1 subtype (B in grey and non-B in black).

HIV-1 subtype distribution was well balanced at baseline between the two treatment groups. For patients on LPV/r monotherapy, the distribution of viral subtype was as follows: 56 B subtype (68%) and 27 non-B subtypes including CRF02_AG 16%, A 2%, G 4% and others subtypes 10%. Figure 1 describes the distribution of gag CS mutations according to HIV subtype. Non-B subtype isolates were significantly more likely to harbour more than two substitutions in p2/NC site (88% vs 32%, p<0.0001), more than three substitutions in the TFPp6pol site (100% vs 54%, p<0.0001) and more than three substitutions in the p6pol site (50% vs 14%, p<0.0001) than B subtype isolates, respectively (Figure 1).

Of note, the level of hairpin free energy was significantly higher in B viruses (median −23.35 kcal/mol, Inter Quartile Range −23.95 to −21.75) compared with non-B viruses (median −20.85 kcal/mol, IQR −22.10 to −20.0) (p = 0.0005).

The impact of baseline substitution in gag CS on subsequent LPV/r single-drug regimen treatment outcome was analyzed. Amino-acid residues G, T, N, P and S at position A374 in the *gag* reading frame tended to predict virological failure (p = 0.053). Substitutions at position A374 were significantly more likely in non-B subtype (70%) versus B subtype viruses (36%, p = 0.005). Amino-acid residues G, I, P and S at position V484 in the *gag-pol* reading frame were significantly associated with virological failure (p = 0.024). Non-B subtype viruses were significantly more likely to harbour substitutions at position V484 (85%) than B subtype viruses (13%, p<0.001). In contrast, amino-acid residues G, N and R at position S451 in *gag* reading frame were significantly associated with virological success (p = 0.026). The presence of more than two substitutions in p2/NC site at baseline significantly predicted virological failure (p = 0.0479). In contrast, the presence of at least three substitutions in the TFPp6pol site or in the p6pol site was not associated with virological failure. Only the presence of substitutions at positions V484 (OR = 4.87 (IQR 1.6–14.8), p = 0.005) and S451 (OR = 0.12 (IQR 0.02–0.6), p = 0.01) remained significantly associated with subsequent virological outcome in multivariate analysis.

No impact of folding and stability of *gag-pol* RNA frameshift on virological response was observed.

### Evolution of gag cleavage site mutations in patients experiencing virological failure

Twenty-three patients experienced virological failure while on LPV/r during the study course. Table S1 focuses on positions at which codons changes where evident at baseline and at the time of virological failure. Compared to baseline sequences, additional substitutions in gag CS were evident in 11/23 patients (in CA/p2 (n = 2), in p2/NC (n = 3), in p1/p6 (n = 2), in TFP/p6pol (n = 3), in p6pol (n = 1)). Reversions to wild type amino-acid residue were observed in 9/23 patients (in CA/p2 (n = 1), in p2/NC (n = 4), in TFP/p6pol (n = 3), in p6pol (n = 1)).

When focusing on gag CS mutations usually detected in treatment-experienced isolates (A431V, K436R, I437V, L449F/V, P452S, P453L/A), the L449F mutation was not detected at baseline and emerged at the time of failure in 2 cases (patients #507, #1401). For these two patients, no minor or major changes in protease gene were evidenced.

PI major resistance mutations were evidenced at the time of virological failure in 5 patients as described previously [26]. Emergence of major PI resistance mutations was associated with concomitant change in gag CS in 3/5 isolates (Table S1, patients #311, #3002, #3103). Baseline number and gag CS mutation was not associated with the selection of additional major PI mutation at virological failure.

## Discussion

The major result of the MONARK trial was that LPV/r monotherapy demonstrated lower rates of virological suppression when compared to LPV/r triple therapy [24]. In addition, long-term 96-week follow-up data are available for patients randomised to first-line LPV/r single drug regimen [25]. Intriguingly, in most patients experiencing virological failure, this was not explained by the emergence of resistance mutations in the protease gene while under protease inhibitor drug-selective pressure [26]. Moreover analysis of predictive factors of virological response in patients randomized to LPV/r single drug regimen indicated that having a plasma HIV-RNA load below 400 copies/ml at week 4 and harbouring an HIV-1 subtype B were independently associated with an increased probability of success [27]. Here we first show that gag CS and the gag-pol frameshift region were highly polymorphic especially in patients infected with a non-B subtype strains. Second, the presence of mutations in gag CS prior to any antiretroviral therapy influences virological outcome of a first-line PI/r single-drug regimen. However, given that (i) the gag substitutions previously showing an association with reduced PI susceptibility in the absence of protease resistance mutations, or reduced susceptibility/increased replicative capacity in their presence are not those showing an association with virological failure in this study, (ii) the study of the gag region in patients undergoing virological failure does not show any significant accumulation of substitutions from baseline and (iii) the baseline substitutions associated with virological failure do not accumulate at failure and are not associated with the emergence of minor or major protease resistance mutations at failure, it might be argued that the highest rate of virological failure was more likely related to suboptimal adherence among patients harbouring a non-B subtype compared to those harbouring a B subtype [27]. Multivariate analysis showed that the detected association between the gag polymorphisms and virological outcome remained independent from patients adherence.

Gag CS and the gag-pol frameshift region were highly polymorphic at baseline in the 82 assessable patients, especially those infected with a non-B subtype strains. The most polymorphic gag CS were the p2/NC site in the *gag* reading frame and both TFP/p6pol and p6pol sites in the *gag-pol* reading frame. Interestingly, the presence of more than two substitutions in p2/NC site at baseline was significantly associated with virological failure, non-B subtype isolates being more likely to harbour more than two substitutions in this specific site. This result brings now some light on our previous finding which suggest that, in spite of potential confounding factor evidenced in this study (adherence and non-B subtype), virological failure appeared significantly more frequent in non-B (46%) than in B subtype isolates (20%, p = 0.0479) [27].

Several studies reported that the p2/NC CS is highly polymorphic [22], [28], [29] with a statistically significant association between these mutations and the development of high-level PI cross-resistance [28]. Indeed, selection of mutation at position 373 in p2/NC correlated with poor virological response in a context of mutated protease [22]. In our study, the presence of mutations at position 374 at baseline tended to predict virological failure. This was true in a context of wild-type protease, suggesting that this mutation might be a first step towards the development of high level resistance if protease-associated mutations were to emerge subsequently.

Recently, the impact of the natural polymorphism in *gag* gene of non-B subtype isolates was evaluated *in vitro* on the drug susceptibility and the catalytic efficiency of the protease ([30], [31]. Introduction of a CRF01_AE-gag/PR region in a background of subtype B pNL4-3 virus (CRF01_AE-gag/PR recombinant) clearly showed that these viruses were significantly less susceptible to 9 PIs than the CRF01_AE-PR-recombinant viruses (without AE gag region) [31], which is consistent with a relevant impact of the polymorphism of CRF01_AE gag on PI susceptibility. In keeping with Jinnopat et al, Gupta et al demonstrated that full-length HIV-1 Gag from A or C subtype HIV-1 strains can contribute to 3 to 14 fold change of reduction in lopinavir susceptibility [30]. Authors concluded that considering the protease gene alone in a genetic background of B subtype may overestimate PI susceptibility.

Finally, in keeping with previous reports [32]–[34], we found that the level of hairpin free energy was higher in B than in non-B subtypes. It has been shown that a decrease in free energy of the RNA secondary structure of the gag-pol frameshift signal induces instability in this signal, poor efficiency of change in the gag-pol open reading frame and a diminution of enzyme production.

*Protease* gene of non-B subtypes displays a high degree of polymorphism that potentially alters the susceptibility of the protease to PIs [35], [36]. Indeed, phenotypic studies revealed that naturally occurring amino acid substitutions found in *protease* gene of non-B subtypes can affect the drug susceptibility of the protease [37]–[39].

Mutations K436R, I437V, L449F and P453L in gag CS, previously evident in treatment-experienced isolates [23], were present at baseline in 13/82 (16%) patients but were not associated with virological failure. Our results are in keeping with Verheyen et al, who showed that the prevalence of K436R, I437V and P453L was higher in antiretroviral-naïve patients infected with non B subtypes than with a B subtype [40]. These polymorphisms observed at positions 436, 437, 449, and 453 might influence the selection of treatment-associated CS substitutions at these positions.

Recently, in the 2IP-ANRS 127 trial evaluating a first-line dual-boosted PI regimen in naïve patients, Larrouy et al demonstrated that the presence of gag CS mutation 128 (p17/p24) and mutation 449 (p1/p6gag) at baseline were associated with subsequent virological failure [33]. We did not find such an association in the Monark trial. Results from 2IP might however hardly be extrapolated to the Monark trial because the PIs used were different (fosamprenavir-atazanavir/r and saquinavir-atazanavir/r versus lopinavir/r) as well as the definition and the time of assessment of virological failure (week 16 in the 2IP trial versus week 96 in the Monark trial). Follow-up was longer in the Monark trial (96 weeks), thus we can not compare directly the impact of the gag region on the selection of PI major mutation between the two studies.

Most virological failures were not associated with specific changes in Gag sequence at the time of failure. Of note, the selection of the mutation L449F was evident at the time of failure in two patients. As previously described, this mutation was observed only in protease inhibitor-experienced patients with protease resistance mutations [22], [23]. L449F was evident in association with the I50V in the *protease* gene, increasing the level of resistance to amprenavir [18], and also to lopinavir [29]. L449F mutation might act as compensatory mutation allowing an increase of the cleavage activity of the mutant protease. In our case, we can speculate that the emergence of the L449F mutation could precede and/or promote the selection of the I50V mutation under LPV/r monotherapy selective pressure. Further phenotypic analysis of clonal isolates harbouring the L449F might help understanding the decreased susceptibility to lopinavir.

Major PI mutations were evident in 5 patients among the 23 experiencing virological failure and studied in the present analysis. There was no consistent association between the emergence of major PI resistance mutations and baseline Gag CS region or changes in Gag sequence at the time of virological failure. We previously reported the selection of the L76V major PI mutation in 3 patients, all three infected with HIV-1 CRF02_AG subtype, confirming that the L76V mutation (+/− the M46I mutation) is a novel resistance pathway emerging during failure on a first line LPV/r-based regimen [41]–[43]. Nijhuis and others suggested that the L76V mutation was associated with the emergence of the A431V mutation in the NC/p1 (p7/p1) site, thus compensating the severe reduction in replicative capacity of the L76V mutant [43]. In contrast to previous reports, the A431V mutation was not selected in our study, even in patients with the L76V mutation at virological failure, which might be due to the CRF02_AG particular genetic background. Of note, mutation A431V might have been selected later in our patients if drug selective pressure was maintained with an ongoing viral replication.

In conclusion, we show that pre-therapy mutations in gag cleavage site sequence were significantly associated with the virological outcome of a first-line LPV/r single drug regimen, in spite of the absence of consistent association with either the emergence of major PI resistance mutations or with changes in gag sequences at the time of virological failure. Gag cleavage site is highly polymorphic in antiretroviral-naive patients harbouring a non-B HIV strain. The non-B subtype may be associated with a high risk of virological failure on first-line LPV/r monotherapy. Our results, together with the similar outcome between B and non-B HIV-1 strains with PI/r-based triple combinations [44], [45] suggest that the impact of mutations in gag CS might be less critical in a context of PI/r-based triple combination (with drugs acting on another target than protease gene) than in a PI/r monotherapy setting. Further studies are warranted to better understand the determinants and prognostic factors of virological outcome of non-B HIV-1 strains in a setting of PI/r monotherapy, especially if the LPV/r monotherapy strategy is proposed as a second-line option after failure on a NNRTI-based first-line triple combination in resource-limited countries [46], [47] where non-B HIV-1 subtypes are predominant.

## Supporting Information

Table S1 GAG cleavage site and protease mutations at screening and at failure in the 23 patients who experienced virological failure.
(DOC)
