# Supplementary material for: Polymorphism in Gag Gene Cleavage Sites of HIV-1 Non-B Subtype and Virological Outcome of a First-Line Lopinavir/Ritonavir Single Drug Regimen
Source: PLoS One. 2011 Sep 20;6(9):e24798. doi: 10.1371/journal.pone.0024798 (PMC3176769; doi:10.1371/journal.pone.0024798)
Supplement: Table S1 — GAG cleavage site and protease mutations at screening and at failure in the 23 patients who experienced virological failure. (DOC) [file pone.0024798.s001.doc]

Table S1

|  |  |  |  | **gag reading frame** | | | | | | | | | | | | | | | | | | |
| --- | --- | --- | --- | --- | --- | --- | --- | --- | --- | --- | --- | --- | --- | --- | --- | --- | --- | --- | --- | --- | --- | --- |
|  |  |  |  | **CA/p2** | | |  | **P2/NC** | | | | | | |  | **NC/p1** | | |  | **p1/p6gag** | | |
|  | **Patient** | **Subtype** | **Visit** | **V362** | **L363** | **A364** |  | **S373** | **A374** | **T375** | **I376** | **M378** | **R380** | **G381** |  | **A431** | **K436** | **I437** |  | **L449** | **S451** | **P453** |
|  | 104 | A | SCREENING |  |  |  |  | deletion | T | N | V |  |  |  |  |  |  |  |  | P |  |  |
|  |  |  | W40 |  |  |  |  | deletion | T | N | V |  |  |  |  |  |  |  |  | P |  |  |
|  | 204 | B | SCREENING |  |  |  |  | deletion | | A | V |  |  |  |  |  |  |  |  |  |  |  |
|  |  |  | W40 |  |  |  |  | deletion | | A/T | V |  |  |  |  |  |  |  |  |  |  |  |
|  | 207 | B | SCREENING |  |  |  |  | P |  |  |  |  |  |  |  |  |  |  |  |  |  |  |
|  |  |  | W24 |  |  |  |  | P |  |  |  |  |  |  |  |  |  |  |  |  |  |  |
|  | 311 | B | SCREENING | V/I |  |  |  | P |  | A |  |  | K |  |  |  |  |  |  |  |  |  |
|  |  |  | W72 |  |  |  |  | P |  | A |  |  | K |  |  |  |  |  |  |  |  |  |
|  | 507 | B | SCREENING |  |  |  |  | A | N | T/A |  |  |  |  |  |  |  |  |  |  |  |  |
|  |  |  | W24 |  |  |  |  | A | N | A |  |  |  |  |  |  |  |  |  | L/F |  |  |
|  | 508 | B | SCREENING |  |  |  |  |  |  |  |  |  |  |  |  |  |  |  |  |  |  |  |
|  |  |  | W96 |  |  |  |  |  |  |  |  |  |  |  |  |  |  |  |  |  |  |  |
|  | 902 | B | SCREENING |  |  |  |  |  |  |  | V |  |  |  |  |  |  |  |  | P |  | T |
|  |  |  | W48 |  |  |  |  |  |  |  | V |  |  |  |  |  |  |  |  | P |  | T/I |
|  | 903 | B | SCREENING |  |  |  |  | S/P | N | N/I |  |  |  |  |  |  | R |  |  |  |  |  |
|  |  |  | W24 |  |  |  |  | S/P | N | N/I |  |  |  |  |  |  | R |  |  |  |  |  |
|  | 1103 | B | SCREENING |  |  |  |  |  |  | A |  |  | R/K |  |  |  |  |  |  |  |  |  |
|  |  |  | W84 | V/I |  |  |  | S/T |  | T/A |  |  |  |  |  |  |  |  |  |  |  |  |
|  | 2111 | B | SCREENING |  |  |  |  |  |  |  | V |  |  |  |  |  |  |  |  |  |  |  |
|  |  |  | W24 |  |  |  |  |  |  |  | V |  |  |  |  |  |  |  |  |  |  |  |
|  | 2116 | B | SCREENING |  |  |  |  |  | T |  |  |  |  |  |  |  |  |  |  |  |  |  |
|  |  |  | W60 |  |  |  |  |  | T |  |  |  |  |  |  |  |  |  |  |  |  |  |
|  | 3204 | B | SCREENING |  |  |  |  |  |  | N | V |  | K |  |  |  |  |  |  |  |  |  |
|  |  |  | W40 |  |  |  |  | S/P |  | N | I/V | M/I | K |  |  |  |  |  |  |  |  |  |
|  | 4004 | C | SCREENING |  |  |  |  |  | T | N |  |  | K | S |  | insertion | |  |  | P |  | L |
|  |  |  | W96 |  |  |  |  |  | T | N |  |  | K | S |  | insertion | |  |  | P |  | L |
|  | 206 | CRF01 | SCREENING |  |  |  |  | T | G | G |  |  |  |  |  |  | R |  |  |  |  |  |
|  |  |  | W64 |  |  |  |  | T | G | G |  |  |  |  |  |  | R |  |  |  |  |  |
|  | 112 | CRF02 | SCREENING |  |  |  |  | Q | N | N | I/V | M/V |  |  |  |  |  |  |  | P |  |  |
|  |  |  | W31 |  |  |  |  | Q | N | N |  |  |  |  |  |  |  |  |  | P |  |  |
|  | 113 | CRF02 | SCREENING |  |  |  |  | Q | P | N |  |  | K |  |  |  |  |  |  | P |  |  |
|  |  |  | W24 |  |  |  |  | Q | P | N |  |  | K |  |  |  |  |  |  | P |  |  |
|  | 310 | CRF02 | SCREENING |  |  |  |  | Q | P/S | N |  |  |  |  |  |  |  |  |  | P |  |  |
|  |  |  | W40 |  |  |  |  | Q | S | N |  |  |  |  |  |  |  |  |  | P |  |  |
|  | 1403 | CRF02 | SCREENING |  |  |  |  | Q | A/T | N | I/V |  |  |  |  |  |  |  |  | P |  |  |
|  |  |  | W26 |  | L/F | A/G |  | Q | A/T | N | I/V |  | R/K |  |  |  |  |  |  | P |  |  |
|  | 1404 | CRF02 | SCREENING |  |  |  |  | Q | N |  |  |  |  |  |  |  |  |  |  | P |  |  |
|  |  |  | W72 |  |  |  |  | Q | N |  |  |  |  |  |  |  |  |  |  | P |  |  |
|  | 3002 | CRF02 | SCREENING |  |  |  |  |  | S | N |  |  |  | N/S/D |  |  |  |  |  | P |  |  |
|  |  |  | W96 |  |  |  |  |  | S | N |  |  |  |  |  |  |  |  |  | P |  |  |
|  | 3103 | CRF02 | SCREENING |  |  |  |  | Q | T | N | V |  |  |  |  |  |  |  |  | P |  |  |
|  |  |  | W62 |  |  |  |  | Q | S | N |  |  |  |  |  |  |  |  |  | P |  |  |
|  | 1401 | G | SCREENING |  |  |  |  | A | insertion | |  |  | K | S |  |  |  |  |  |  | N |  |
|  |  |  | W80 |  |  |  |  | A | insertion | |  |  | K | S |  |  |  |  |  | F | N |  |
|  | 4201 | G | SCREENING |  |  |  |  | A | T |  | insertion | | K | G/S |  |  |  |  |  |  | N | L |
|  |  |  | W60 |  |  |  |  | A | A/T |  | insertion | | K |  |  |  |  |  |  |  | N | L |

**Table S1-continued**

|  |  |  |  | **gag-pol reading frame** | | | | | | | | | | | | | |  | |  | | **PROTEASE** |  |
| --- | --- | --- | --- | --- | --- | --- | --- | --- | --- | --- | --- | --- | --- | --- | --- | --- | --- | --- | --- | --- | --- | --- | --- |
|  |  |  |  | **TFP/p6pol** | | | | | | |  | **p6pol** | | | | |  | | **Free energy** |  | | **new IAS minor and major** | |
|  | **Patient** | **Subtype** | **Visit** | **R435** | **E436** | **D437** | **F440** | **L441** | **G443** | **K444** |  | **V484** | **S485** | **F486** | **N487** | **F488** |  | | **Kcal/mol** | | **mutations at failure** | | |
|  | 104 | A | SCREENING |  |  | N |  | Q |  | E |  | P | insertion | | S |  |  | | -20,80 |  | |  |  |
|  |  |  | W40 |  |  | N |  | Q |  | E |  | P | insertion | | S |  |  | | -20,80 |  | |  |  |
|  | 204 | B | SCREENING |  |  |  |  | P |  | E |  | I |  |  |  | L |  | | -23,80 |  | |  |  |
|  |  |  | W40 |  |  |  |  | P |  | E |  | I |  |  |  | L |  | | -23,80 |  | |  |  |
|  | 207 | B | SCREENING |  |  | N |  | P | R/G |  |  |  |  | L | S |  |  | | -22,20 |  | |  |  |
|  |  |  | W24 |  |  | N |  | P | R |  |  |  |  | L | S |  |  | | -22,20 |  | |  |  |
|  | 311 | B | SCREENING |  |  | N |  | P |  | E |  | I |  | C | S |  |  | | -25,10 |  | |  |  |
|  |  |  | W72 |  |  |  |  | P |  | E |  | T |  | C |  |  |  | | -25,20 |  | | 10F, 82A |  |
|  | 507 | B | SCREENING |  |  | D/N |  | P |  | E |  |  |  | L | D |  |  | | -22,90 |  | |  |  |
|  |  |  | W24 |  |  | D/N |  | P |  | E |  |  |  | L | D |  |  | | -22,80 |  | |  |  |
|  | 508 | B | SCREENING |  |  |  |  | P |  | E |  |  | insertion | | S |  |  | | -23,80 |  | |  |  |
|  |  |  | W96 |  |  |  |  | P |  | E |  |  | insertion | | S |  |  | | -23,80 |  | |  |  |
|  | 902 | B | SCREENING |  |  | N |  |  |  | E/K |  |  |  | L |  |  |  | | -23,80 |  | |  |  |
|  |  |  | W48 |  |  | N | C | P |  | E |  |  |  | L |  |  |  | | -23,90 |  | |  |  |
|  | 903 | B | SCREENING |  |  | N |  | P |  |  |  |  |  |  | S |  |  | | -26,30 |  | |  |  |
|  |  |  | W24 |  |  | N |  | P |  |  |  |  |  |  | S |  |  | | -26,30 |  | | **46I** |  |
|  | 1103 | B | SCREENING |  |  | N |  | Q | R/G | E |  |  |  | L | D |  |  | | -20,50 |  | |  |  |
|  |  |  | W84 |  |  | N |  | Q | R/G | E |  |  |  | L | D |  |  | | -19,70 |  | |  |  |
|  | 2111 | B | SCREENING |  |  | N |  | P |  | E/K |  |  |  | L |  |  |  | | -22,90 |  | |  |  |
|  |  |  | W24 |  |  | N |  | P |  | E/K |  |  |  | L |  |  |  | | -22,90 |  | | 13V, 60E, 64V, 69K, 70R | |
|  | 2116 | B | SCREENING |  |  | N |  | Q | R |  |  |  |  | L | S |  |  | | -20,20 |  | |  |  |
|  |  |  | W60 |  |  | N |  | Q | R |  |  |  |  | L | S |  |  | | -20,20 |  | |  |  |
|  | 3204 | B | SCREENING |  |  |  |  | P |  | E |  |  |  |  | S |  |  | | -23,80 |  | |  |  |
|  |  |  | W40 |  |  | D/N |  | P |  | E |  |  |  |  | S |  |  | | -23,80 |  | |  |  |
|  | 4004 | C | SCREENING |  |  | N |  | P |  | E |  | G | T | L |  | C |  | | -25,10 |  | |  |  |
|  |  |  | W96 |  |  | N |  | P |  | E |  | G | T | L |  | C |  | | -25,10 |  | |  |  |
|  | 206 | CRF01 | SCREENING |  |  | N |  | Q | R/G | E |  | S |  | L | S |  |  | | -18,20 |  | |  |  |
|  |  |  | W64 |  |  | N |  | Q |  | E |  | S |  | L | S |  |  | | -18,10 |  | | 10F |  |
|  | 112 | CRF02 | SCREENING |  | K/E | N |  | Q |  |  |  | I | insertion | |  |  |  | | -20,00 |  | |  |  |
|  |  |  | W31 |  |  | N |  | Q |  |  |  | I | insertion | |  |  |  | | -21,30 |  | |  |  |
|  | 113 | CRF02 | SCREENING |  |  | N |  | Q | R | E |  | I |  |  | S |  |  | | -21,50 |  | |  |  |
|  |  |  | W24 |  |  | N |  | Q | R | E |  | I |  |  | S |  |  | | -21,50 |  | |  |  |
|  | 310 | CRF02 | SCREENING |  |  | N |  | Q | R | E |  | I | insertion | |  |  |  | | -20,90 |  | |  |  |
|  |  |  | W40 |  |  | N |  | Q | R | E |  | I | insertion | |  |  |  | | -20,90 |  | | **76V** |  |
|  | 1403 | CRF02 | SCREENING |  |  | N |  | Q |  | E |  | S |  |  |  |  |  | | -23,20 |  | |  |  |
|  |  |  | W26 |  |  | N |  | Q |  | E |  | S |  |  |  |  |  | | -23,20 |  | |  |  |
|  | 1404 | CRF02 | SCREENING |  |  | N |  | Q |  | E |  | I | insertion | L |  |  |  | | -21,90 |  | |  |  |
|  |  |  | W72 |  |  | N |  | Q |  | E |  | I | insertion | L |  |  |  | | -21,90 |  | |  |  |
|  | 3002 | CRF02 | SCREENING |  |  | N |  | Q |  | E |  |  | insertion | | S |  |  | | -20,80 |  | |  |  |
|  |  |  | W96 |  |  | N |  | Q |  | E |  |  | insertion | | S |  |  | | -20,80 |  | | **76V** |  |
|  | 3103 | CRF02 | SCREENING |  |  | N |  | Q |  | E |  | I | insertion | |  |  |  | | -20,80 |  | |  |  |
|  |  |  | W62 |  |  | N |  | Q |  | E |  | I | insertion | |  |  |  | | -23,20 |  | | **46I, 76V** |  |
|  | 1401 | G | SCREENING |  |  | N |  | Q |  | E |  | I |  |  | S |  |  | | -17,10 |  | |  |  |
|  |  |  | W80 | R/K |  | N |  | Q |  | E |  | I |  |  | S |  |  | | -17,10 |  | |  |  |
|  | 4201 | G | SCREENING |  |  | N |  | Q |  | E |  | deletion | |  | S | L |  | | -18,00 |  | |  |  |
|  |  |  | W60 |  |  | N |  | Q |  | E |  | deletion | |  | S | L |  | | -19,70 |  | |  |  |
